# Accumulation and Tissue Distribution of Dinophysitoxin-1 and Dinophysitoxin-3 in the Mussel *Crenomytilus grayanus* Feeding on the Benthic Dinoflagellate *Prorocentrum foraminosum*

**DOI:** 10.3390/md15100330

**Published:** 2017-10-24

**Authors:** Polina A. Kameneva, Ekaterina A. Krasheninina, Valentina V. Slobodskova, Sergey P. Kukla, Tatiana Yu. Orlova

**Affiliations:** 1 National Scientific Center of Marine Biology, A.V. Zhirmunsky Institute of Marine Biology, Far Eastern Branch of the Russian Academy of Sciences, ul. Palchevskogo 17, Vladivostok 690041, Russia; ekaterina_krasheninina@mail.ru (E.A.K.); torlova06@mail.ru (T.Y.O.); 2 Far Eastern Federal University, School of Natural Sciences, ul. Sukhanova 8, Vladivostok 690950, Russia; 3 V.I. Il’icev Pacific Oceanological Institute, Far Eastern Branch of the Russian Academy of Sciences, ul. Baltiyskaya 43, Vladivostok 690041, Russia; slobodskova@list.ru (V.V.S.); kukla.sp@mail.ru (S.P.K.)

**Keywords:** dinophysistoxin-1, dinophysistoxin-3, *Prorocentrum foraminosum*, *Crenomytilus grayanus*, DSTs accumulation, alkaline comet assay, genotoxicity

## Abstract

A DTX-1-producing microalga, *Prorocentrum foraminosum*, from Peter the Great Bay, Sea of Japan, was fed to Gray’s mussels, *Crenomytilus grayanus*, for 12 days. An increase in DTX-1 and 7-*O*-acyl-DTX-1 (DTX-3) was observed in the digestive gland, kidneys, and gills. The digestive gland accumulated 91–100% of DTX-1 + DTX-3; and kidneys and gills accumulated, up to 8.5% and 4.3%, respectively. The kidneys had a distinctive pattern of toxin accumulation where the concentration of DTX-1 did not grow significantly after the eighth day of feeding, indicating the potential of DTX-1 elimination. The digestive gland and gills predominantly accumulated DTX-1, with a dramatic increase between Days 8 and 12. The DTX-3 content was highest in the digestive gland. The composition of DTX-3 in the acyl groups was similar for the digestive gland and kidneys, and did not change during feeding. The total toxin uptake of mussels exceeded the total toxin content from ingested cells by 2.4 times, showing that toxins may have accumulated from the seawater. This assumption needs to be further proved. The muscle, gonads, and mantle remained free of toxins. No genotoxic effect was observed in the gills and digestive gland.

## 1. Introduction

Diarrheic shellfish toxins (DSTs) belong to a group of lipophylic metabolites, associated with dinoflagellates of the genera Prorocentrum [1] and Dinophysis [2]. There are three main toxins that represent DSTs: okadaic acid (OA), dinophysistoxin-1 (DTX-1), and dinophysistoxin-2 (DTX-2). All of these compounds are specific inhibitors of serine/treonine phosphatases [3,4], enzymes involved in the regulation of various processes in eukaryotic cells [5]. They have been shown to cause diarrheic syndrome in mammals after the consumption of contaminated shellfish [6,7]; to disturb DNA structure and cell cycle regulation, to have a negative effect on the immune and nervous systems [8]. Accordingly, these compounds are called free and active forms.

While feeding on toxin-producing dinoflagellates, bivalve mollusks can accumulate DSTs in their soft tissues. Each of the main compounds can be transformed into 7-*O*-acyl derivatives in bivalves [9], and are collectively called dinophysistoxin-3 (DTX-3). Acyl chain decreases the affinity of toxin to their target proteins [10]. Accordingly, these compounds are known as conjugated and non-active forms. DTX-3 is included in the DST group as they become toxic after the hydrolysis of acyl chain [7,11,12]. The role of transforming the active toxin to DTX-3 is supposed to protect bivalves from DSTs activity [13]. However, these protective mechanisms may not always be effective, as it has recently been shown that a high density of *Prorocentrum lima* may cause oxidative DNA damage in haemocytes of the mussel *Mytilus galloprovincialis* [14].

It has been shown that the main DSTs accumulate in the digestive gland of bivalve, and can be partially transformed into DTX-3 derivatives [9,15,16,17,18,19,20]. However, controversy remains on the distribution of DSTs in the tissues of bivalves, and the ratios of active and non-active compounds. In some species, the digestive gland is apparently the main organ for toxin accumulation [21,22]; in other species, a significant part of toxins can be stored in the gonads, mantle, and other organs [18,23]. The dynamics of toxin accumulation in different organs of bivalves still needs to be clarified. Therefore, for the first time, we investigated the dynamics of DTX-1 and DTX-3 accumulation and tissue distribution in *C. grayanus* in controlled feeding conditions. To achieve this goal, adult mussels were fed with cultured *Prorocentrum foraminosum*, a DTX-1-producing dinoflagellate [24,25]. The concentration of DTX-1 and DTX-3 was measured in samples of the digestive gland, gills, kidneys, muscle, and gonads with the mantle of each mussel. To reveal possible negative impacts of DSTs on bivalves, an alkaline comet assay of the digestive gland and gill tissue was applied. In this article, the abbreviation DSTs is used to report the sum of DTX-1 and DTX-3, as OA and DTX-2 were not observed in either the culture of *P. foraminosum*, or in the tissues of *C. grayanus*.

## 2. Results

### 2.1. Diarrhetic Shellfish Toxins in C. grayanus during Acclimatization

*C. grayanus* mussels were collected in Peter the Great Bay, Sea of Japan in September 2016, and underwent an acclimatization process to minimize the influence of possible toxin accumulation from the natural environment. Prior to acclimatization, the mussels contained DSTs in the digestive gland, kidneys, and gills. The digestive gland contained an average of 127.5 ± 48.6 ng/g (mean ± SD; *n* = 3) of total DSTs. The kidneys and gills contained trace amounts of DTX-3, based on a comparison of hydrolyzed and non-hydrolyzed samples.

After acclimatization, the concentration of DSTs in the digestive gland and kidneys did not change significantly (*p* > 0.05). Furthermore, the gills of most mussels became free of toxins. The initial toxin content in these organs was taken into account for all of the toxin concentration calculations.

### 2.2. DSTs during Feeding on C. muelleri (Control Study)

The control experiment to investigate the initial toxin concentration and possible toxin elimination was set by feeding *C. grayanus* with *Chaetoceros muelleri*, a non-toxin-producing diatom. The initial density of *C. muelleri* in the tanks was 1708.2 ± 371.1 × 10^3^ cells/L (mean ± SD; *n* = 11). At the end of each day, no cells of *C. muelleri* were observed.

The average concentration of total DSTs in the digestive gland during control feeding was 194.4 ± 78.8 ng/g (mean ± SD; *n* = 9). The kidneys of most mussels became toxin free by Day 12, and the gills were free of toxins.

### 2.3. Dinophysistoxin-1 Exposure

Cultures of *P. foraminosum* contained DTX-1 in cells and cell-free media at a concentration of 1.9 ± 0.6 pg/cell and 43.8 ± 15.9 ng/mL, respectively (mean ± SD; *n* = 11). The average cell density in the cultures was 9.6 ± 2.4 × 10^3^ cells/mL (mean ± SD; *n* = 12). After 24 h of feeding, the concentration of microalgae in the tanks was 23 ± 16 × 10^3^ cells/L (mean ± SD; *n* = 12). Table 1 shows the detailed feeding of the mussels during the 12 days.

The total exposure to DTX-1 on the fourth, eighth, and twelfth days of feeding was calculated based on the values of toxin concentration in cultures, the volume of the cultures added, and the total number of ingested *P. foraminosum* cells (Table 2).

### 2.4. DSTs during Feeding on P. foraminosum

Feeding with *P. foraminosum* led to an increase of DST content in the digestive gland, kidneys, and gills. The digestive gland accumulated 91–100% of total DSTs; and in the kidneys and gills, up to 8.5% and 4.3% of DSTs, respectively. The muscle, gonads, and mantle remained free of DSTs during the 12 days of feeding. 

**Figure 1:**
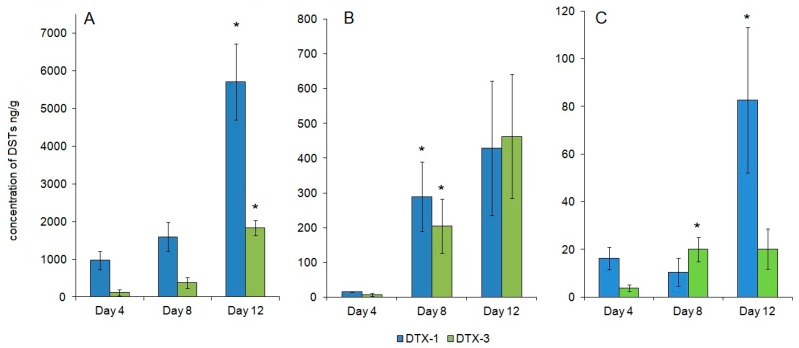
Dinophysistoxin-1 (DTX-1) and Dinophysistoxin-3 (DTX-3) concentration in the digestive gland (**A**); kidneys (**B**); gills (**C**) of mussels fed on *P. foraminosum.* Value is mean ± SE; *n* = 3. Mann-Whitney *p* value < 0.05 is indicated by the asterisk.

The DTX-1 and DTX-3 content of the digestive gland, kidneys, and gills on the fourth, eighth, and twelfth days of feeding are shown in Figure 1.

The total DSTs in the digestive gland reached 999.8 ± 271.6 ng/g (mean ± SE; *n* = 3) by the fourth day. In the kidneys and gills, the toxin content at this stage was similar and much lower than in the digestive gland at 21.4 ± 4.1 ng/g and 18.2 ± 2.3 ng/g (mean ± SE; *n* = 3), respectively. The major toxin form was DTX-1 in all of the organs.

In the digestive gland and gills, a slight increase of DST concentration was noticed on the eighth day. In the kidneys, the concentration of DTX-1 and DTX-3 increased dramatically by the eighth day (Figure 1B). DTX-3 was the major form of toxin in the gills (65%) at this stage. In the digestive gland and kidneys, this form accounted for 19% and 41%, respectively.

A dramatic growth of DSTs was noticed in the digestive gland and gills by the twelfth day of feeding. The final concentration of DSTs in these organs was 6838.5 ± 651.1 ng/g and 92.5 ± 14.7 ng/g (mean ± SE; *n* = 3), respectively. The highest concentration of DTX-3 among all of the organs was observed in digestive gland on the twelfth day of feeding 1835.5 ± 197.7 ng/g (mean ± SE; *n* = 3), which accounted for 27% (Figure 1A). In the gills, the concentration of DTX-3 remained the same, but due to the accumulation of DTX-1, the percentage of DTX-3 (% of total) decreased to 22% (Figure 1C). No significant change of DST concentration was noticed in the kidneys after the eighth day. The final concentration of DSTs in this organ was 724.7 ± 312.0 ng/g (mean ± SE; *n* = 3). DTX-3 in this organ accounted for 52% on the twelfth day (Figure 1B). 

The composition of the DTX-3 acyl chains was determined in the digestive gland and kidneys of mussels fed on *P. foraminosum* (Table 3). DTX-3 from gills were not analyzed for their acyl chains profile due to the low concentration.

The composition of the acyl chains of DTX-3 did not change during feeding and was similar for both the digestive gland and kidneys. The major acyl chains found in DTX-3 were C16:0, C16:1, and C18:1. The minor acyl chains C14:0, C18:0, and C18:2 were found in both the digestive gland and kidneys. C15:0, C18:3, and C18:4 were found only in the digestive gland and proportion of DTX-3 with each of these acyl chains was lower than 1.5%. All of the corresponding fatty acids were found in both cells of *P. foraminosum* and the lipid fractions from the digestive gland of *C. grayanus*. C15:0 was found only in the lipid fractions from the digestive gland of *C. grayanus,* where it accounted for less than 1%.

The total toxin accumulation by each mussel fed on *P. foraminosum* for 12 days was calculated as a sum of the DST content in the digestive gland, gills, and kidneys; other organs were considered to have zero toxin concentration. The DST content was the concentration of DSTs in each organ (µg/g) multiplied by the wet weight of the organ (g) (Table 4). A high variability in toxin accumulation was observed between individuals.

### 2.5. Alkaline Comet Assay

**Figure 2:**
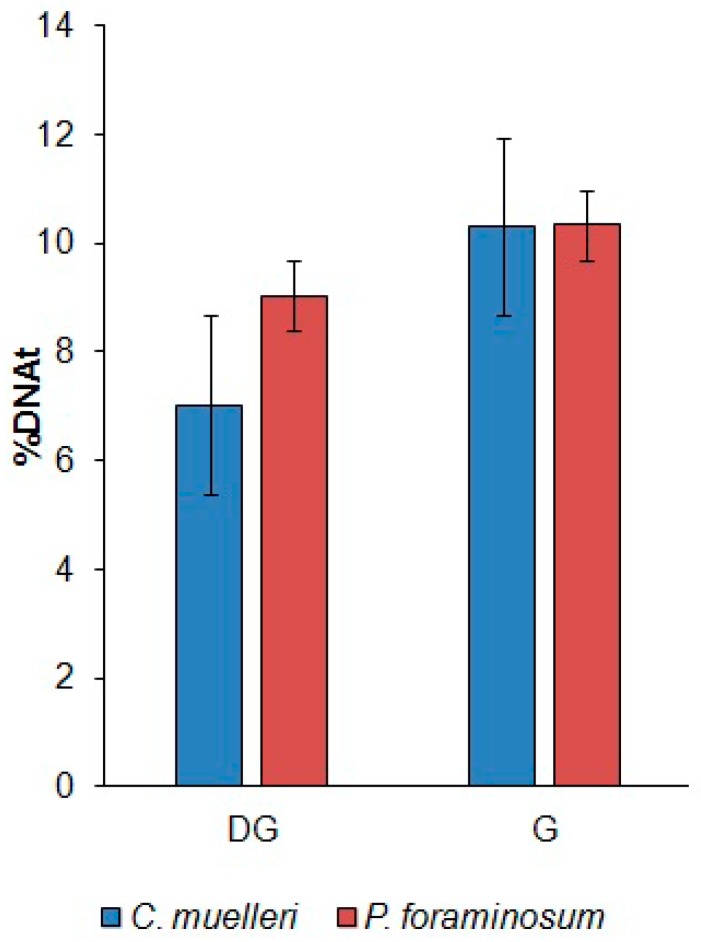
Quantification of DNA damage in the cells of the mussels’ gills and digestive gland on Day 12 of feeding with *C. muelleri* and *P. foraminosum* (using the alkaline comet assay). %tDNA, percentage of DNA in the tail of comet; DG, digestive gland; G, gills.

The quantity of DNA strand breaks was represented by a % of DNA in the tail (% tDNA) for 150 comets in each sample. For both the experimental and control groups, the level of DNA strand breaks was not higher than 12%. The cells of the digestive gland of the mussels fed on *C. muelleri* and *P. foraminosum* had 7.02 ± 0.63 %tDNA and 9.03 ± 0.49 %tDNA, respectively (mean ± SD; *n* = 150). The percentage of DNA strand breaks in the gills was the same for both the *C. muelleri* and the *P. foraminosum* diets (Figure 2).

## 3. Discussion

### 3.1. Acclimatization and Feeding with C. muelleri

Mussels collected from their natural habitat in September 2016 contained a moderate concentration of DTX-1, which is typical for Peter the Great Bay, Sea of Japan, in this season of year [26]. Prior to acclimatization, the digestive gland, kidneys, and gills all contained DSTs. The major part of the toxins was stored in the digestive gland.

*C. muelleri* (clone MBRU_CM_88) was used as food during the acclimatization and control feeding experiment. All of the *C. muelleri* cells added to the mussels were filtered within a 24-h period, meaning that the mussels successfully fed on them. Feeding with *C. muelleri* for seven days during acclimatization led to toxin elimination from the gills of most mussels, but not from the kidneys and digestive gland. Feeding with *C. muelleri* for 12 days during the control experiment led to toxin elimination from the kidneys of most mussels. However, 12 days of a *C. muelleri* diet was not enough to remove toxins from the digestive gland of *C. grayanus*.

### 3.2. P. foraminosum as a Source of DXT-1 and the Exposure to DXT-1

*P. foraminosum* (clone MBRU_PrRUS_7) cultured in laboratory conditions is capable of producing DTX-1 [24], which is one of the main compounds of DSTs. Other DSTs were not found in the culture of this *P. foraminosum* clone. DTX-1 was found in the cells of *P. foraminosum* and in the cell-free culture media. The exposure of mussels to DTX-1 was continuous, as indicated by the *P. foraminosum* cells found in the seawater within 24 h after the addition of microalgae (Table 1). Thus, *C. grayanus* was exposed to two types of DTX-1: DTX-1 from the cells, and DTX-1 from seawater (Table 2).

### 3.3. DSTs Accumulation

The digestive gland, kidneys, and gills were the only organs where DSTs were observed during 12 days of feeding with *P. foraminosum*. Twelve days of exposure to high concentrations of DTX-1 was not enough for DSTs to appear in the muscle, gonads, and mantle. A major portion of DSTs accumulated in the digestive gland, suggesting that this was the main organ of toxin accumulation in *C. grayanus*. The digestive gland proved to be the main organ for DSTs accumulation in most studies [18,21,27,28,29]. 

The dynamics of toxin accumulation was different for the digestive gland and gills in comparison with the kidneys. In the kidneys, the highest toxin increase of both free and conjugated forms was observed between Days 4 and 8. After the eighth day, the concentration of the free form of toxins did not change significantly. The slight increase of DST content in this organ was due to the accumulation of conjugated forms. Our hypothesis is that the kidneys may not be able to accumulate high concentrations of free DSTs due to its potential role in eliminating toxins. The potential for mussels to eliminate toxins in free form was also discussed for *M. edulis* fed on *D. acuta* [30]. In the digestive gland and gills, the highest DST increase was observed between Days 8 and 12 due to the increased concentration of DTX-1. This can be explained by the continuous uptake of DTX-1 from food with a limited capacity to excrete and transform it into conjugated forms. Mussels are known to less efficiently transform DSTs to DTX-3 when compared to other bivalves [19].

The composition of the acyl chains in DTX-3 was very similar for the digestive gland and kidneys, suggesting that DTX-3 may form in one organ with the following distributions to other organs. The digestive gland was the most likely to be the organ of DTX-3 formation given that was where the majority of DTX-3 was found. Moreover, the in vitro acylation of OA and DTX-1 in the presence of the extract from the digestive gland of *Mizuhopecten yessoensis*, but not from the other organs, have been shown by Konoki et al. [13]. The presence of DTX-3 with acyl chains, which corresponded to the fatty acid found only in lipid fraction of bivalves (C15:0) suggested that the fatty acids of mussels are more important than the fatty acids of microalgae for the formation of DTX-3. Acyl chains with chain lengths more than 18 carbon atoms and with more than two double bonds were not found in DTX-3, despite the fact that the concentrations of C20:5 and C22:6 were higher than C16:0, C16:1, and C18:1 each (Table 3). Similar results on the fatty acid profile of DTX-3 were reported for naturally contaminated scallop *P. yessoensis* and mussel *Mytilus coruscus* from Japan [31]. However, the acyl chains of C20:5 and C22:6 were found in DTX-3 of *M. galloprovincialis* and *Donax trunculus* [32].

If only the DTX-1 content of cells (Table 2) was considered for the estimation of total toxin uptake, then by the twelfth day, mussels accumulated 240% of DTX-1 (Table 4). The other source of toxin in this study was the dissolved toxin from the culture media. When both the DTX-1 from cells and dissolved DTX-1 were considered in the calculations, then by the twelfth day, mussels accumulated 57% of the DTX-1 provided. Previous studies of the controlled feeding of bivalves with DST-producing dinoflagellates showed a total toxin accumulation ranging from 1% to 75% of toxin exposure [23,30,33], but the dissolved toxins were not measured during the accumulation process. The proportion of toxins acquired from the cells and from the seawater is unknown. The assumption that mussels are able to acquire toxins directly from seawater requires further investigation.

### 3.4. Influence of DTX-1 on DNA Damage

The alkaline comet assay was used in this study to determine the amount of DNA damage in the gills and digestive gland cells after 12 days of exposure to *P. foraminosum* cultures.

The observed levels of DNA strand breaks were considered as low. Similar levels of DNA strand breaks have been observed in similar research in the control group, meaning that this level of DNA strand breaks is considered normal [14]. The obtained results revealed the absence of significant levels of DNA damage in *C. grayanus* gills and digestive gland cells after 12 days of feeding on *P. foraminosum*, despite the fact that the active forms of DTX-1 were present at a high concentration. These results show the inability of the *P. foraminosum* clone MBRU_PrRUS_7 to cause a genotoxic effect during 12 days of exposure. A similar study of *M. galloprovincialis*, exposed to a high density of *P. lima*, showed a positive correlation of DNA damage to the concentration of dinoflagellates after 48 h of exposure [14]. The observed difference may be explained by the presence of different DSTs in cultures of *P. lima* and *P. foraminosum.*

## 4. Materials and Methods

### 4.1. Reagents

Standard solutions of okadaic acid, dinophysistoxin-1, dinophysistoxin-2, and certified reference material of mussel’ tissue were purchased from the Certified Reference Material Program (CRMP) of the Institute for Marine Biosciences, National Research Council (Ottawa, ON, Canada). A K medium kit (50 L) was purchased from the Provasoli-Guillard National Center for Marine Algae and Microbiota NCMA. Methanol hypergrade for liquid chromatography-mass-spectrometry (Merck, Darmstadt, Germany), acetonitrile for high-performance liquid-chromatography, far UV, gradient grade (J.T.Baker, Deventer, The Netherlands), monensin sodium salt (90–95% for thin layer chromatography), and reagents for alkaline comet assay were purchased from Merck, Darmstadt, Germany. Formic acid ~98% for mass-spectrometry and NH_4_OH ≥ 25% in H_2_O were obtained from Fluka analytical. NaOH and HCl were analytical grade.

### 4.2. Culturing of Microalgae

*P. foraminosum* (MBRU_PrRUS_7) was cultured using K enriched seawater medium made from autoclaved seawater with 34‰ salinity. For feeding, 24 flasks (150 mL) containing 100 mL of culture media were set. Culture incubation took place in an incubation chamber KBW 400 (Binder, Tuttlingen, Germany) at 20 °C with a 12:12 h light:dark cycle and illumination of 39 μmol m^−2^·s^−1^ for eight weeks.

*C. muelleri* (MBRU_CM_88) was cultured using K enriched seawater medium made from autoclaved seawater with 34‰ salinity. For acclimatization, seven flasks containing 150 mL of culture were set. For feeding, 24 flasks containing 100 mL of culture media were set. Incubation of cultures was in an incubation chamber KBW 400 (Binder, Tuttlingen, Germany) at 20 °C with a 12:12 h light:dark cycle and illumination of 39 μmol m^−2^·s^−1^ for 1 week. *C. muelleri* cultures had a density of 435.1 ± 106.8 × 10^3^ cells/mL and were free of toxins.

### 4.3. Cell Number Counting

For cell number estimation, the cultures of *P. foraminosum* and *C. muelleri* were well-mixed in a culture flask. Culture (1 mL) was removed directly from the flasks, 4 mL of cell-free K medium was added to the sample for dilution, and then fixed with a 0.2% *v*/*v* acidic Lugol’s solution. Counting was done under the light microscope; a Sedgewick-Rafter chamber was used for *P. foraminosum*, and a haemocytometer for *C. muelleri*. Appropriate dilution factors were included in the calculations.

For cell number estimation in tanks, after a 24-h period of feeding, 500 mL of seawater was concentrated by reverse filtration using a 2 µm nucleopore filter to get the 5–15 mL sample. It was fixed with a 0.2% *v*/*v* acidic Lugol’s solution and counted as described for the cultures.

### 4.4. Mussel Acclimatization

A total of 40 mussels were collected in Peter the Great Bay, Sea of Japan, in late September (usually when the lowest concentration of naturally accumulated DSTs is observed [26]) and were placed in tanks with 50 L of filtered (with a 1 µm flow-through filter) and UV-treated seawater. The water in the tanks was constantly aerated, and the temperature was maintained at 16 °C. The mussels were fed daily with 150 mL of *C. muelleri* culture reaching the initial concentration of 1.740.6 ± 427.3 × 10^3^ cells/L. The water was changed daily. Mussel survival was estimated by their ability to react to touch and to keep their shell closed.

### 4.5. Mussel Feeding

After acclimatization, the mussels were divided into six tanks (five mussels in each) with 9 L of filtered (with a 1 µm flow-through filter), UV-treated, and constantly aerated seawater. In three tanks, the mussels were fed with *P. foraminosum*; in the other three tanks used *C. muelleri* as a control. Microalgae were added once a day after a complete water change during the 12 days. Mussels’ sampling was made on Days 4, 8, and 12 of feeding by removing mussels from one of the tanks.

### 4.6. Sampling of Mussels

The following samples were taken from three random mussels (five mussels for the 12th day) from each of the tanks: the digestive gland, muscle, kidneys, gills, mantle, and gonads. Mussels were opened by cutting the adductor muscle, rinsed with distilled water, placed on the ice, and organs were separated with a blade. The excess water was removed by placing the organs on filter paper; the organs were weighed, placed in separate zip-lock plastic bags, and frozen at −20 °C before extraction.

### 4.7. Toxin Extraction 

#### 4.7.1. Mussels’ Tissues

DTX-1 extraction was made following the harmonized procedure [34] with few changes. A sub-sample of each organ was weighed, homogenized, and supplemented with 90% methanol at a ratio of 1:10 in two portions. After each addition of methanol, the mixture was vortexed for 2 min and centrifuged for 10 min at 3000 *g*. The methanol portions were combined.

#### 4.7.2. Algal Cells

Forty mL of well-mixed culture were transferred to a 50 mL screw-cup tube and centrifuged at 4000 *g* for 30 min. Supernatant containing almost no cells was transferred to a clean tube and 1.5 mL of 90% methanol was added to the pellet. The pellet was sonicated for 5 min in an ice bath and vortexed for 1 min, followed by centrifugation at 4000 *g* for 10 min. The supernatant was transferred to a glass tube with a screw cup. The residue was extracted again with 1 mL of 90% methanol; the extracts were then combined and adjusted to 3 mL by 90% methanol.

#### 4.7.3. Media

40 mL of media were filtered through the syringe-driven filter 0.45 nm (Merck, Darmstadt, Germany) for the removal of the microalgae cells. Media samples were loaded onto a solid phase extraction (SPE) cartridge (Supelco, Ballefonte, PA, USA) containing 0.3 mg of C-18 sorbent previously conditioned with methanol and distilled water. DSTs were eluted by 3 mL of methanol.

#### 4.7.4. Toxin Hydrolysis

The hydrolysis of the conjugated forms of the DSTs was done as described earlier [16]. A 500 µL portion of the extract was placed in a glass vial, supplemented with 100 µL of 2.5 M NaOH, and incubated at 76 °C for 40 min. The samples were placed on ice immediately after treatment, and 100–120 µL of 2.5 M HCl was added to stop the hydrolysis. The pH was checked using indicator paper. The samples were filtered before injection to a high-performance liquid-chromatograph.

### 4.8. High-Performance Liquid Chromatography Mass-Spectrometric Analysis of DTX-1

High-performance liquid chromatography mass-spectrometric analysis of DTX-1 in all of the samples was performed on a high-performance liquid chromatography instrument Prominence LC-20AD (Shimadzu, Kyoto, Japan), equipped with autosampler (SIL-20A) (Shimadzu, Kyoto, Japan), and a high-resolution mass-spectrometer IT-TOF (Shimadzu, Kyoto, Japan) with an electrospray ionization module. For separation, a reversed phase C18 column (Shodex, Showa Denko, Tokyo, Japan; 150 × 2.1 mm, 5 µm) equipped with a guard column was used. The HPLC instrument parameters were as follows: column oven, 40 °C; flow rate, 0.2 mL/min; and binary gradient elution was applied. Mobile phase A was as follows: deionized H_2_O + 50 mM NH_4_ in form of (NH_4_OH) + 2 mM formic acid; mobile phase B: 95% acetonitrile + 50 mM NH_4_ in the form of (NH_4_OH) + 2 mM formic acid. The chromatographic program was as follows: start with 35% of B, gradient to 95% of B for 8 min, followed by gradient to 100% of B for 15 min, followed by 100% of B for 38 min. Column re-equilibration between injections was 8 min. A sample portion of 5 µL was used for injection. The MS parameters were as follows: CDL temperature, 240 °C; heat block, 200 °C; nebulizing gas flow, 1.5 L/min; capillary voltage, 3.5 kV; ion accumulation time for all *m/z* ranges 100 ms; detector voltage, 1.6 kV. Mass-spectrometric detection was done in selected ion monitoring in negative ionization mode for the following *m*/*z* ranges: 669–670 for detection of internal standard, and 803–818 for detection of DSTs.

### 4.9. Using Monensin as an Internal Standard

**Figure 3:**
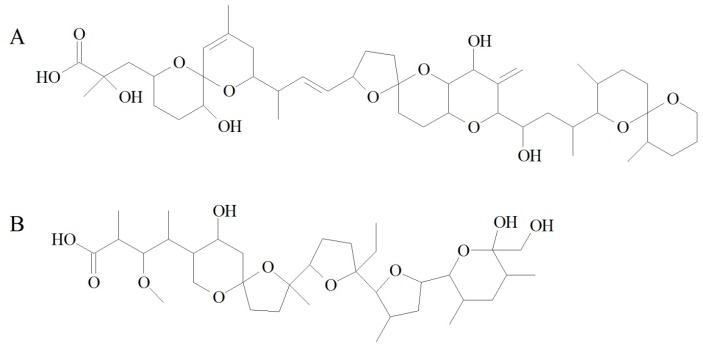
Chemical structures of main DTX-1 (**A**), and monensin (**B**).

Monensin has some structural similarities with the free forms of DSTs [35] (Figure 3). It is considered to be suitable as an internal standard.

The validation analysis was performed using the certified standard reference material, the results of which are presented in Table 5. No significant difference between the certified and measured concentrations of DTX-1 was found.

The relative peak area and concentration had a linear relationship in the range of 1.52–192.0 ng/mL of DTX-1 or monensin. A level of 15.2 ng/g in tissue was considered as the limit of quantification, and 2 ng/g was the limit of detection in this study.

### 4.10. Quantitative DTX-1 Calculations 

Monensin was used as an internal standard to estimate possible loses during extraction and hydrolysis. Monensin solution in methanol (7.4 µg/mL) was added to each sample with the volume of 10 µL per 1 mL of extract in the beginning of the extraction process. Estimation of the toxin concentration was based on the equation:

C_(Monensin)_/S_(Monensin)_ = C_(toxin)_/S_(toxin)_

where C is the concentration of monensin or toxin in the sample; and, S is the area of the peak in the chromatogram. Blank analysis was as follows: monensin solution in 90% methanol was prepared and underwent all of the extraction manipulations each time to estimate the extraction efficiency.

The total toxin concentration was calculated for organs based on their wet weight; for cell pellet, based on number of cells in the sample; and, for the medium, based on the volume of the medium tested. Statistical analysis of the data was based on the Mann-Whitney non-parametric test using the free MS Excel build-on program Real Stat.

### 4.11. HPLC-MS Analysis of DTX-3

HPLC-MS analysis of DTX-3 was performed on a high-performance liquid chromatography instrument Prominence LC-30AD (Shimadzu, Kyoto, Japan), equipped with autosampler (SIL-30A) (Shimadzu, Kyoto, Japan), and a triple quadrupole mass-spectrometer LCMS-8060 (Shimadzu, Kyoto, Japan) with electrospray ionization module. For separation, the reversed phase cartridge C18 column (YMC ProC18, 10 × 4 mm, 5 µm) was used. The HPLC instrument parameters were as follows: column oven, 40 °C; flow rate, 0.2 mL/min; and binary gradient elution was applied. Mobile phase A was as follows: deionized H_2_O + 50 mM NH_4_ in form of (NH_4_OH) + 2 mM formic acid; mobile phase B: 95% acetonitrile + 50 mM NH_4_ in form of (NH_4_OH) + 2 mM formic acid. The chromatographic program was as follows: start with 20% of B, gradient to 100% of B for 10 min, and hold 100% of B for 30 min. The column re-equilibration between injections was 10 min. A sample portion of 5 µL was used for injection. The MS parameters were as follows: Desolvation line temperature, 250 °C; heat block, 400 °C; nebulizing gas flow, 2 L/min; interface voltage, 3.0 kV; dwell time for all *m/z* ranges 100 ms. Mass-spectrometric detection was done in negative ionization MRM mode for the following *m*/*z* transitions: 14:0-DTX-1, 1027.7 > 227.2; 15:0-DTX-1, 1041.7 > 241.2; 16:0-DTX-1, 1055.7 > 255.2; 17:0-DTX-1, 1069.7 > 269.3; 18:0-DTX-1, 1083.7 > 283.3; 20:0-DTX-1, 1111.7 > 311.2; 22:0-DTX-1, 1139.8 > 339.3; 14:1-DTX-1, 1025.7 > 225.2; 16:1-DTX-1, 1053.7 > 253.2; 18:1-DTX-1, 1081.7 > 281.3; 20:1-DTX-1, 1109.8 > 310.3; 18:2-DTX-1, 1079.7 > 279.2; 18:3-DTX-1, 1077.7 > 277.2; 18:4-DTX-1, 1075.7 > 275.2; 18:5-DTX-1, 1073.7 > 273.2; 20:4-DTX-1, 1103.7 > 303.2; 20:5-DTX-1, 1101.7 > 301.2; 22:6-DTX-1, 1127.7 > 327.2. Collision energy for all transitions was −70 v. Additionally, a scan event in negative ion operation mode was added to each analysis at *m*/*z* range 800–1200.

### 4.12. Lipids Extraction

Prior to the extraction, *P. foraminosum* cells were filtered on the GF/C glass microfiber filter (45 mm; Whatman, Maidstone, UK). A filter was placed in a glass vial with a screw cap. The digestive gland of *C. grayanus* was homogenized and an aliquot was placed in a glass vial with the screw cap. Lipid extraction was done according to the procedure of Bligh and Dyer [36].

After extraction, the separation of polar and non-polar lipid fractions from the digestive gland of *C. grayanus* was made on solid phase extraction silica gel cartridges that were conditioned with chlorophorm. Neutral lipids were eluted by chlorophorm, and polar lipids were eluted by chlorophorm:methanol (1:2, v:v).

### 4.13. Analysis of Fatty Acids Composition of P. foraminosum and C. grayanus

Fatty acids were analyzed as methyl esters by gas chromatography with a flame ionization detector. Methyl esters were synthesized by the transmethylation of total lipids or lipid fractions dissolved in hexane with 2% H_2_SO_4_ in methanol during 1.5 h at 90 °C. Gas chromatography was performed on a Shimadzu GC-2010, equipped with a Supelcowax column (30 m × 0.25 mm × 0.25 µm) in isothermal mode. Evaporator temperature = 240 °C, column temperature = 205 °C, and detector temperature = 250 °C. Helium was used as a carried gas with the linear velocity—35 cm/s. Fatty acids were identified by use of equivalent chain length (ECL) values [37].

### 4.14. Isolation of Gill and Digestive Gland Cells for Alkaline Comet Assay

Experiments to study the damage of DNA structure caused by DSTs were performed on the gill and digestive gland cells of *C. grayanus.* Slime was removed from the gills and digestive gland by triple washing with a cold (4 °C) Ca^2+^ and Mg^2+^ free isotonic solution (500 mM NaCl, 12.5 mM KCl, 5 mM EDTA-Na_2_, and 20 mM Tris-HCl, pH 7.4). The tissues were gently cut into small pieces with scissors and placed in 4–5 mL of isotonic solution. After 30–40 min incubation, the cells detached from tissues were removed from the gill and digestive gland fragments by filtering through a 40 μm sieve. Cells in the filtrate were precipitated by centrifugation and re-suspended in isotonic solution to a concentration of 105 cells/mL.

### 4.15. Alkaline Comet Assay

The alkaline comet assay [38], modified to be applied to marine organisms [39], was used for the bivalve gill and digestive gland cell suspensions to determine the level of DNA strand breakage. A portion of 50 μL of cell suspension was supplemented with 100 μL of 1% low-melting point agarose (LKB, Bromma, Sweden) in 0.04 M phosphate buffer (pH 7.4) at 37 °C, thoroughly mixed, placed on a glass slide coated with 1% agarose for better adhesion, and covered with a cover glass. The sample was placed for 3 min in a fridge for a gel to form. The cover glass was carefully removed; each slide was immersed for 1 h into a lysing solution (2.5 M NaCl; 0.1 M EDTA-Na^2^, 1% Triton X-100; 10% DMSO; 0.02 M Tris, pH 10), and placed in darkness. After washing with cold distilled water, the slides were transferred to an electrophoresis buffer (300 mM NaOH and 1 mM EDTA-Na^2^), and kept for 40 min. Electrophoresis was carried out at 2 V/cm for 20 min. After neutralization (0.4 M Tris-HCl, pH 7.4), the slides were stained with ethidium bromide (2 μg/mL). The DNA comets were visualized and registered using a scanning fluorescence microscope (Zeiss, AxioImager A1, Carl Zeiss AG, Oberkochen, Germany) equipped with an AxioCam MRc digital camera, Carl Zeiss AG, Oberkochen, Germany. Digital images were processed using the Casp 1.2.2 software (CASPlab, Wroclaw, Poland), which allowed for the calculation of various parameters of comets to estimate the level of DNA damage. In the control and experimental groups of bivalves, five slides each containing no less than 100 comets were analyzed. For each experiment, the results were statistically evaluated by comparing the mean group parameters (*p* < 0.05) of DNA damage in both groups of bivalves using the nonparametric Kruskal-Wallis test.

## 5. Conclusions

This study shows the dynamics of DTX-1 and DTX-3 accumulation and distribution in certain organs of the mussel *C. grayanus* fed on benthic dinoflagellate *P. foraminosum*. The digestive gland was confirmed to be the main organ for DST accumulation in *C. grayanus* (91–100% of the total toxin content), the kidneys and gills contained lower amounts of toxins. DTX-3 was observed in all organs subject to toxin accumulation, but with different proportions. The toxin accumulation pattern of the digestive gland and gills in comparison with the kidneys were different, indicating the potential ability of the kidneys to eliminate DTX-1. In the digestive gland and gills, the main toxin form in the end of the feeding was DTX-1. The composition of the acyl chains in DTX-3 from the digestive gland and kidneys was very similar and did not change during feeding. An assumption that mussels may be able to accumulate toxins directly from the seawater was made as the total toxin uptake of mussels exceeded the total toxin content from the ingested microalgal cells. No genotoxic effect was observed in the gills and digestive gland of the mussels fed on *P. foraminosum* for 12 days, despite the high concentration of the free form of DTX-1 in these organs.

## Figures and Tables

**Table 1:** Feeding of mussels *C. grayanus* with *P. foraminosum*.

Days of Feeding	Culture Volume (mL)	Cell Density (×10^3^ Cells/mL)	Total Cells Added (×10^3^ Cells)	Initial Cell Density in Tanks (×10^3^ Cell/L)	Cell Density in Tanks after 24 h (×10^3^ Cells/L)	Total Cells Ingested (×10^3^ Cells)
Day 1	90	9.1	820.4	91.2	10.5	726.1
Day 2	95	6.9	655.5	72.8	4.3	616.5
Day 3	100	9.6	963.3	107.0	7.0	900.1
Day 4	102	10.2	1041.1	115.7	16.7	891.3
Day 5	100	10.7	1067.3	118.6	54.8	574.0
Day 6	100	5.9	588.0	65.3	19.2	415.6
Day 7	100	10.2	1015.0	112.7	24.7	792.1
Day 8	100	7.5	750.0	83.3	65.8	157.7
Day 9	80	11.6	925.6	102.8	0	925.6
Day 10	87	8.2	716.6	79.6	25.4	488.3
Day 11	85	12.7	1082.3	120.3	24.7	860.0
Day 12	90	6.1	551.4	61.2	23.0	344.3

**Table 2:** Total exposure to Dinophysistoxin-1.

Source of DTX-1	Day 4	Day 8	Day 12
DTX-1 from cells (µg)	5.95	9.63	14.61
DTX-1 from media (µg)	16.95	34.47	49.50

**Table 3:** Acyl chain composition of DTX-3 (% of total) in the digestive gland and kidneys of *C. grayanus* fed on *P. foraminosum* and corresponding fatty acids composition (% of total) of the total lipids of *P. foraminosum* and lipid fractions of the *C. grayanus* digestive gland.

Acyl Group	Acyl Chain of DTX-3 ^1^		Fatty Acids (FA) ^2^		
	Digestive Gland	Kidney	*P. foraminosum*	*C. grayanus* Digestive Gland Neutral Lipids	*C. grayanus* Digestive Gland Polar Lipids
14:0	6.15 ± 1.47	5.12 ± 0.76	11.09	9.90	11.52
15:0	1.33 ± 0.67	nd	nd	0.73	0.29
16:0	34.98 ± 2.01	38.49 ± 0.74	16.02	15.46	12.59
17:0	nd	nd	nd	1.57	1.78
18:0	3.31 ± 0.71	3.76 ± 1.06	1.38	2.27	15.27
20:0	nd	nd	0.19	0.10	0.05
22:0	nd	nd	nd	0.02	nd
14:1	nd	nd	0.29	0.26	0.07
16:1 *	27.65 ± 4.96	25.34 ± 2.39	2.90	16.32	1.96
18:1 *	25.66 ± 4.32	26.37 ± 4.18	23.74	6.31	2.58
20:1	nd	nd	nd	3.36	8.49
18:2 *	2.50 ± 1.05	1.90 ± 0.00	0.17	2.67	0.61
18:3	0.36 ± 0.00	nd	1.84	0.93	0.37
18:4	0.20 ± 0.00	nd	4.17	0.39	0.15
18:5	nd	nd	2.50	nd	nd
20:4	nd	nd	nd	1.94	4.74
20:5	nd	nd	12.38	20.60	8.42
22:6	nd	nd	18.62	6.26	5.43
Other	na	na	4.71	10.93	19.16

nd—not detected, na—not applicable. ^1^ Value is mean % ± SD, for digestive gland, *n* = 7; for kidneys, *n* = 2. ^2^ Value is mean %, *n* = 2, SD is not reported, but no more than 10%. * multiple peaks were found, which probably corresponds to multiple isomers. Data are presented as a sum of peak areas. Other: minor fatty acids were observed in the FA profiles of total lipids of *P. foraminosum* (12:0, *iso*-16:0, 16:3, 21:5, 24:0 and 26:0) and lipid fractions of *C. grayanus* (19:0, 17:1, 19:1, 20:2, 22:2, 20:3, 22:5).

**Table 4:** DSTs content in organs (µg/organ) of *C. grayanus* fed on *P. foraminosum* for 12 days.

Organ	Mussel 1	Mussel 2	Mussel 3	Mussel 4	Mussel 5
Digestive gland	9.679	13.306	5.245	5.811	1.229
Kidneys	0.064	0.905	0.026	0.181	0.002
Gills	0.060	0.012	0.062	0.060	0.023
Total per mussel (µg)	9.803	14.223	5.333	6.052	1.254
∑ total of mussels (µg)	-	-	-	-	36.665

**Table 5:** Results of monensin validation.

Compound	Certified Concentration	Measured Concentration
Active forms		
Okadaic acid	1.07 ± 0.08	1.039
Dinophysistoxin-1	1.07 ± 0.11	0.963
Dinophysistoxin-2	0.86 ± 0.08	0.699
Active+conjugated forms		
Okadaic acid	2.4	2.450
Dinophysistoxin-1	1.1	0.998
Dinophysistoxin-2	2.2	2.123

## References

[1] Hoppenrath M., Chomerat N., Horiguchi T., Schweikert M., Nagahama Y., Murray S. (2013). Taxonomy and phylogeny of the benthic *Prorocentrum* species (Dinophyceae)-A proposal and review. Harmful Algae.

[2] Reguera B., Velo-Suarez L., Raine R., Park M.G. (2012). Harmful *Dinophysis* species: A review. Harmful Algae.

[3] Takai A., Murata M., Torigoe K., Isobe M., Mieskes G., Yasumoto T. (1992). Inhibitory effect of okadaic acid derivatives on protein phosphatases. A study on structure-affinity relationship. Biochem. J..

[4] Bialojan C., Takai A. (1988). Inhibitory effect of a marine-sponge toxin, okadaic acid, on protein phosphatases. Biochem. J..

[5] Ceulemans H., Bollen M. (2004). Functional diversity of protein phosphatase-1, a cellular economizer and reset button. Physiol. Rev..

[6] Yasumoto T., Murata M., Oshima Y., Sano M., Matsumoto G.K., Clardy J. (1985). Diarrhetic shellfish toxins. Tetrahedron.

[7] Vale P., Sampayo M.A.D.M. (2002). First confirmation of human diarrhoeic poisonings by okadaic acid esters after ingestion of razor clams (*Solen marginatus*) and green crabs (*Carcinus maenas*) in Aveiro lagoon, Portugal and detection of okadaic acid esters in phytoplankton. Toxicon.

[8] Valdiglesias V., Prego-Faraldo M.V., Pasaro E., Mendez J., Laffon B. (2013). Okadaic Acid: More than a diarrheic toxin. Mar. Drugs.

[9] Marr J.C., Hu T., Pleasance S., Quilliam M.A., Wright J.L.C. (1992). Detection of new 7-*O*-acyl derivatives of diarrhetic shellfish poisoning toxins by liquid chromatography-mass spectrometry. Toxicon.

[10] McNabb P., Botana L. (2008). Chemistry, metabolism and chemical analysis of okadaic acid group toxins. Seafood and Freshwater Toxins.

[11] Vale P., Antónia M., Sampayo M. (1999). Esters of okadaic acid and dinophysistoxin-2 in Portuguese bivalves related to human poisonings. Toxicon.

[12] Rodríguez I., Alfonso A., Antelo A., Alvarez M., Botana L.M. (2016). Evaluation of the impact of mild steaming and heat treatment on the concentration of okadaic acid, dinophysistoxin-2 and dinophysistoxin-3 in mussels. Toxins.

[13] Konoki K., Onoda T., Watanabe R., Cho Y., Kaga S., Suzuki T., Yotsu-Yamashita M. (2013). *In vitro* acylation of okadaic acid in the presence of various bivalves’ extracts. Mar. Drugs.

[14] Prego-Faraldo M.V., Valdiglesias V., Laffon B., Mendez J., Eirin Lopez J.M. (2016). Early genotoxic and cytotoxic effects of the toxic dinoflagellate *Prorocentrum lima* in the mussel *Mytilus galloprovincialis*. Toxins.

[15] Rossignoli A.E., Fernandez D., Regueiro J., Marino C., Blanco J. (2011). Esterification of okadaic acid in the mussel *Mytilus galloprovincialis*. Toxicon.

[16] Suzuki T., Ota H., Yamasaki M. (1999). Direct evidence of transformation of dinophysistoxin-1 to 7-*O*-acyl-dinophysistoxin-1 (dinophysistoxin-3) in the scallop *Patinopecten yessoensis*. Toxicon.

[17] MacKenzie L., Holland P., McNabb P., Beuzenberg V., Selwood A., Suzuki T. (2002). Complex toxin profiles in phytoplankton and Greenshell mussels (*Perna canaliculus*), revealed by LC-MS/MS analysis. Toxicon.

[18] Vale P., Sampayo M.A. (2002). Esterification of DSP toxins by Portuguese bivalves from the Northwest coast determined by LC-MS—A widespread phenomenon. Toxicon.

[19] Torgersen T., Sandvik M., Lundve B., Lindegarth S. (2008). Profiles and levels of fatty acid esters of okadaic acid group toxins and pectenotoxins during toxin depuration. Part II: Blue mussels (*Mytilus edulis*) and flat oyster (*Ostrea edulis*). Toxicon.

[20] Moroño A., Arévalo F., Fernández M.L., Maneiro J., Pazos Y., Salgado C., Blanco J. (2003). Accumulation and transformation of DSP toxins in mussels *Mytilus galloprovincialis* during a toxic episode caused by *Dinophysis acuminata*. Aquat. Toxicol..

[21] Blanco J., Mariño C., Martín H., Acosta C.P. (2007). Anatomical distribution of diarrhetic shellfish poisoning (DSP) toxins in the mussel *Mytilus galloprovincialis*. Toxicon.

[22] Marcaillou C., Haure J., Mondeguer F., Courcoux A., Dupuy B., Pénisson C. (2010). Effect of food supply on the detoxification in the blue mussel, *Mytilus edulis*, contaminated by diarrhetic shellfish toxins. Aquat. Living Resour..

[23] Bauder A.G., Cembella A.D., Bricelj V.M., Quilliam M.A. (2001). Uptake and fate of diarrhetic shellfish poisoning toxins from the dinoflagellate *Prorocentrum lima* in the bay scallop *Argopecten irradians*. Mar. Ecol. Prog. Ser..

[24] Kameneva P.A., Efimova K.V., Rybin V.G., Orlova T.Y. (2015). Detection of dinophysistoxin-1 in clonal culture of marine dinoflagellate *Prorocentrum foraminosum* (Faust M.A., 1993) from the Sea of Japan. Toxins.

[25] Selina M.S. (2017). Morphology and seasonal dynamics of the potentially toxic microalga *Prorocentrum foraminosum* Faust 1993 (Dinophyta) in Peter the Great Bay, the Sea of Japan. Russ. J. Mar. Biol..

[26] Orlova T.Y., Kameneva P.A., Stonik I.V., Morozova T.V., Efimova K.V., Moore L., Eberhart B.-T.L., Wells M.L., Trainer V.L. (2015). Diarrhetic shellfish toxins in Primorsky Krai, Russia. J. Shellfish Res..

[27] Yasumoto T., Oshima Y., Yamaguchi M. (1978). Occurrence of a new type of shellfish poisoning in the Tohoku district. Bull. Jpn. Soc. Sci. Fish.

[28] Prassopoulou E., Katikou P., Georgantelis D., Kyritsakis A. (2009). Detection of okadaic acid and related esters in mussels during diarrhetic shellfish poisoning (DSP) episodes in Greece using the mouse bioassay, the PP2A inhibition assay and HPLC with fluorimetric detection. Toxicon.

[29] Hess P., Nguyen L., Aasen J., Keogh M., Kilcoyne J., McCarron P., Aune T. (2005). Tissue distribution, effects of cooking and parameters affecting the extraction of azaspiracids from mussels, *Mytilus edulis*, prior to analysis by liquid chromatography coupled to mass spectrometry. Toxicon.

[30] Nielsen L.T., Hansen P.J., Krock B., Vismann B. (2016). Accumulation, transformation and breakdown of DSP toxins from the toxic dinoflagellate *Dinophysis acuta* in blue mussels, *Mytilus edulis*. Toxicon.

[31] Suzuki T., Kamiyama T., Okumura Y., Ishihara K., Matsushima R., Kaneniwa M. (2009). Liquid-chromatographic hybrid triple-quadrupole linear-ion-trap MS/MS analysis of fatty-acid esters of dinophysistoxin-1 in bivalves and toxic dinoflagellates in Japan. Fish. Sci..

[32] Vale P. (2006). Detailed profiles of 7-*O*-acyl esters in plankton and shellfish from the Portuguese coast. J. Chromatogr. A.

[33] Matsushima R., Uchida H., Nagai S., Watanabe R., Kamio M., Nagai H., Kaneniwa M., Suzuki T. (2015). Assimilation, accumulation, and metabolism of dinophysistoxins (DTXs) and pectenotoxins (PTXs) in the several tissues of japanese scallop *Patinopecten yessoensis*. Toxins.

[34] European Union Reference Laboratory for Marine Biotoxins (2015). EU-Harmonised Standard Operating Procedure for Determination of Lipophilic Marine Biotoxins in Molluscs by LC-MS/MS.

[35] Cembella A.D. (2003). Chemical ecology of eukaryotic microalgae in marine ecosystems. Phycologia.

[36] Bligh E.G., Dyer W.J. (1959). A rapid method of total lipids extraction and purification. Can. J. Biochem. Physiol..

[37] Christie W.W. (1988). Equivalent chain-lengths of methyl ester derivatives of fatty acids on gas chromatography A reappraisal. J. Chromatogr. A.

[38] Singh N.P., McCoy M.T., Tice R.R., Scheider E.L. (1988). A simple technique for quantification of low levels of DNA damage in individual cells. Exp. Cell Res..

[39] Slobodskova V.V., Solodova E.E., Slinko E.N., Chelomin V.P. (2010). Evaluation of the genotoxicity of cadmium in gill cells of the clam *Corbicula japonica* using the comet assay. Russ. J. Mar. Biol..

